# SLT-VEGF Reduces Lung Metastases, Decreases Tumor Recurrence, and Improves Survival in an Orthotopic Melanoma Model

**DOI:** 10.3390/toxins2092242

**Published:** 2010-08-27

**Authors:** Rachel Ackerman, Joseph M. Backer, Marina Backer, Sini Skariah, Carl V. Hamby

**Affiliations:** 1 Department of Microbiology and Immunology, New York Medical College, Valhalla, NY 10595, USA; Email: rb.ackerman@gmail.com (R.A.); sini_skariah@nymc.edu (S.S.); 2 SibTech, Inc., Brookfield, CT 06804, USA; Email: jbacker@sibtech.com (J.M.B); mbacker@sibtech.com (M.B.)

**Keywords:** biological therapeutics, Shiga-like toxin, SLT-VEGF, melanoma, angiogenesis, metastasis, VEGF receptor targeting

## Abstract

SLT-VEGF is a recombinant cytotoxin comprised of Shiga-like toxin (SLT) subunit A fused to human vascular endothelial growth factor (VEGF). It is highly cytotoxic to tumor endothelial cells overexpressing VEGF receptor-2 (VEGFR-2/KDR/Flk1) and inhibits the growth of primary tumors in subcutaneous models of breast and prostate cancer and inhibits metastatic dissemination in orthotopic models of pancreatic cancer. We examined the efficacy of SLT-VEGF in limiting tumor growth and metastasis in an orthotopic melanoma model, using NCR athymic nude mice inoculated with highly metastatic Line IV Cl 1 cultured human melanoma cells. Twice weekly injections of SLT-VEGF were started when tumors became palpable at one week after intradermal injection of 1 × 10^6^ cells/mouse. Despite selective depletion of VEGFR-2 overexpressing endothelial cells from the tumor vasculature, SLT-VEGF treatment did not affect tumor growth. However, after primary tumors were removed, continued SLT-VEGF treatment led to fewer tumor recurrences (*p* = 0.007), reduced the incidence of lung metastasis (*p* = 0.038), and improved survival (*p* = 0.002). These results suggest that SLT-VEGF is effective at the very early stages of tumor development, when selective killing of VEGFR-2 overexpressing endothelial cells can still prevent further progression. We hypothesize that SLT-VEGF could be a promising adjuvant therapy to inhibit or prevent outgrowth of metastatic foci after excision of aggressive primary melanoma lesions.

## 1. Introduction

The role of angiogenesis in supporting tumor growth and metastasis is well established [1]. Tumors have been shown to secrete pro-angiogenic molecules that induce vascular growth and allow tumor cells to gain access to nutrients as well as provide cellular escape routes. Vascular endothelial growth factor (VEGF) is a key stimulator of angiogenesis, whose receptors, particularly VEGFR-2, are overexpressed in endothelial cells in tumor vasculature. Once proangiogenic molecules, like VEGF, are no longer balanced out by endogenous anti-angiogenic molecules, an angiogenic “switch” occurs, which initiates the vascular phase of tumor growth [2]. Because of the prominence of VEGF/VEGFR signaling, the majority of approved and experimental anti-angiogenesis therapeutics target VEGF and VEGFR-2 [3,4]. Such targets seemed particularly attractive for long term treatment since, unlike cancer cells, endothelial cells comprising the tumor vasculature lack the genetic instability that allows drug resistance to develop [5].

The clinical experience with VEGF/VEGFR inhibitors has been discouraging since, although many types of tumors respond well to these inhibitors initially, they all rapidly develop resistance and patients almost invariably relapse within one year of starting treatment [6,7]. The resistance to anti‑angiogenic drugs appears to be associated with mechanism(s) of their action. Although inhibitors of VEGF/VEGFR signaling were expected to inhibit tumor growth via inhibition of endothelial cell proliferation, experimental evidence and clinical observations indicate that it induces actual vascular regression, most likely by inhibiting pro-survival VEGF functions [8,9]. Vascular regression, in turn, leads to hypoxia in tumors and upregulation of VEGF production, which stimulates tumor revascularization after either prolonged exposure to the drugs or during interruptions in treatment [10]. Surprisingly, revascularization appears to involve endothelial cells that are resistant to VEGF/VEGFR inhibitors through mechanisms that are not fully understood. These may include reliance on alternative signaling pathways or development of resistance to the employed drugs [6].

An alternative approach to anti-angiogenic therapy is to subvert VEGFR for selective delivery of highly cytotoxic agents into endothelial cells, with the expectation that only tumor endothelial cells overexpressing such receptors will internalize therapeutically significant amounts of VEGFR-targeted cytotoxins. Since alternative signal transduction pathways cannot prevent or reverse the cytotoxic activity of plant or bacterial toxins, several groups have used VEGF for targeting such toxins to tumor vasculature [11]. One such protein is SLT-VEGF, which is comprised of human VEGF121 fused to SLT, a site-specific N-glycosidase from Shiga-like toxin [12]. Upon internalization, the SLT moiety cleaves off adenosine 4324 in 28S rRNA, which prevents the ribosome from properly interacting with elongation factors thus impeding protein synthesis and eventually leading to cell death. SLT-VEGF is highly cytotoxic to VEGFR-2 overexpressing cells *in vitro*, and selectively depletes such cells in tumor vasculature of autologous mouse 4T1 mammary carcinoma tumors and of human PC3 prostate tumor xenografts. It inhibits primary tumor growth in these models and also inhibits metastatic dissemination in two orthotopic pancreatic tumor models [11,13].

Judging by the mechanism of SLT-VEGF action, its efficacy should depend on the contribution of VEGFR-2 overexpressing cells to the survival and growth of primary tumors and metastatic lesions at various stages of tumor development through the course of treatment. In this study, we explored the effects of SLT-VEGF on development of primary tumors, and on tumor recurrence and metastatic dissemination after primary tumor excision, using an orthotopic xenograft model of highly metastatic melanoma tumors derived from Line IV Cl 1 human melanoma cells [14,15]. We reasoned that such a model would be relevant to the clinical setting of human melanoma patients undergoing primary tumor excision who are at risk of local tumor recurrence and/or development of distant metastases.

## 2. Materials and Methods

### 2.1. Cultured Cells

Line IV Cl 1 human melanoma cells were a gift from Dr. B.C. Giovanella (The Cancer Research Laboratory, St. Joseph Hospital, Houston, TX). Orthotopic tumors derived from these cells have a high metastatic potential in nude mice as previously shown by Heim *et al.* [14] and confirmed by Hamby *et al.* [15]. They were maintained in RPMI-1640 (Lonza, Walkersville, MD) with 10% bovine growth serum (BGS)(HyClone, Logan, Utah). Porcine aortic endothelial (PAE) cells were obtained from the American Type Tissue Collection (Rockville, MD) and PAE cells stably transfected with human KDR (PAE/KDR) were derived as described [16]. The latter cell lines were maintained in DMEM (Lonza, Walkersville, MD) with 10% BGS. All cell lines were incubated at 37 °C in a 5% CO_2_ atmosphere.

### 2.2. Research Reagents

SLT-VEGF was constructed, expressed and purified as described previously [12] and supplied by SibTech, Inc. Purified human IFN-α was obtained from the NIAID Reference Reagent Repository administered by KamTek, Inc., in Gaithersburg, MD. The lyophilized powder was reconstituted in sterile injection saline and stored in aliquots in vapor phase liquid nitrogen.

### 2.3. Growth Inhibition Assay

Target cells were seeded in triplicate at 1 × 10^3^ cells/well in 96 well plates (Falcon, Becton Dickinson, Franklin Lakes, NJ). The cells were allowed to attach for 24 hours before different concentrations of SLT-VEGF were added to the cultures. After 3 days incubation at 37 °C in 5% CO_2_, 20 μL/well of CellTiter 96^®^ AQueous One Solution Reagent (Promega, Madison, WI) was added to plates and allowed to incubate for 1 hour. The color change was read at 490 nm on a 96 well plate reader (Bio-Rad, Hercules CA, USA). The percent growth inhibition at each concentration of SLT‑VEGF was calculated by comparison to untreated control wells.

### 2.4. Caspase Assays

PAE/KDR cells and Line IV Cl 1 human melanoma cells were plated in 8 well chamber slides (BD Falcon, Franklin Lakes, NJ) at 3 × 10^4^ cells per chamber in 200 µL of DMEM with 10% BGS. The cells were allowed to attach overnight and treated the next day with 26 nM SLT-VEGF. At 18 and 22 hours after addition of SLT-VEGF, the media was removed and 100 µL of a 30× FLICA™ working solution from Vybrant^®^ FAM Caspase Kits (Molecular Probes, Carlsbad, CA) specific for caspase 8 or caspases 3 and 7 were added to slide chambers according to the manufacturer’s instructions. The fluoromethyl ketone moiety of these reagents covalently binds to cysteine residues of activated caspases in cells and can be visualized by the carboxyfluorescein (FAM) reporter. A 2.85 μg/mL solution of 4',6-diamidino-2-phenylindole (DAPI, Sigma-Aldrich, St. Louis, MO) was added to stain cell nuclei. The chamber apparatus was then removed from the slide and coverslips were mounted with Vectashield medium (Vector Laboratories, Burlingame, CA). The slides were analyzed on a Zeiss Axiovert 200 microscope (Carl Zeiss, Thornwood, NY) equipped with appropriate filters and Axiovision Rel 4.5 image capture software. Images of fluorescently stained cells viewed through a 10X objective were captured from three random fields in each treatment well. The percentage of FAM‑stained cells per field was calculated by counting the total number of FAM-positive cells in each field and dividing by the total number of DAPI-stained nuclei in the same field. All caspase experiments were repeated three times.

### 2.5. Human Melanoma Xenograft Studies

New York Medical College institutional policy conforms to the U.S. Public Health Service *Policy on Humane Care and Use of Laboratory Animals* and all animal protocols were reviewed and approved by the institutional use and care of animals committee before experiments were begun. One million Line IV Cl 1 melanoma cells were inoculated intradermally into the flanks of either NCR athymic mice or Balb/c nu/nu mice obtained from the NIH production facility (Frederick, MD). SLT‑VEGF treatment was started when tumors became palpable, which occurred at one and four weeks after tumor cell implantation in NCR athymic and Balb/c nu/nu hosts, respectively. Details of the treatment regimens are given in the Results section. The length, width, and height in mm of each tumor was measured weekly up to and including the date of tumor excision. Tumor volumes were estimated by calculating the average radius *r* from length, width and height measurements and substituting into the formula, V = 2/3πr^3^, for the volume of a hemisphere. Tumor growth rates were expressed as the slopes of linear regression curves calculated from the square roots of tumor volumes plotted against time. Transformation of tumor volumes in this manner yielded regression curves with straight line relationships whose slopes, therefore, provide robust estimates of tumor growth rates. In each study skin tumors were completely excised between six and seven weeks after they first became palpable and following a two week recovery period mice received five additional doses of SLT-VEGF on schedules detailed in the Results. The mice were followed until they showed signs of morbidity or until the conclusion of the study at 22 weeks post tumor cell inoculation.

### 2.6. Lung Histology

At sacrifice, lungs from mice in each study were perfused with formalin before being removed from the thoracic cavity. They were fixed overnight in 10% buffered formalin before being transferred to 75% ethanol for storage. Paired left and right lungs from each mouse were processed for histological staining at either Albert Einstein College (Bronx, NY) or AML laboratories (Frederick, MD) where they were embedded in paraffin blocks and 3 micron-thick sections were cut and stained with hematoxylin and eosin. Slides were analyzed under a light microscope equipped with an ocular gradicule (Olympus America, Inc, Medville, NY). The smallest diameter of each metastatic focus was measured with the five by five grid of the gradicule in which the sides of the individual small squares represent a distance of 0.261 mm at the 40× magnification used to view the slides. The number and diameters of all tumor foci in left and right lung sections of each animal were recorded and analyzed for statistical differences between treatment and control groups.

### 2.7. Immunofluorescence Microscopy

Harvested tumors were frozen in Optimal Tissue Cutting (OCT) compound and then cut on a cryotome into 7 µm sections and mounted on Plus slides (Fisher Scientific, Fair Lawn, NJ) for staining and analysis. We employed a previously published procedure for dual fluorescent immunostaining of VEGFR2 and CD31 [17]. Briefly, sections were blocked in normal rabbit serum and incubated with a primary rat, anti-mouse CD31 antibody (BD Biosciences San Jose, CA), then with a biotinylated rabbit anti-rat secondary antibody (Vector Labs, Burlingame, CA). CD31 staining was visualized by tyramide amplification with Alexa Fluor-594 TSA kits (Molecular Probes Carlsbad, CA) according to the manufacturer’s instructions. Excessive horseradish peroxidase activity was quenched by incubation with 5% H2O2 for 1 hour at RT, followed by incubation with rat, anti-mouse Flk-1(VEGFR-2) antibody (BD Biosciences). After washing and incubation with biotinylated rabbit anti-rat secondary antibody, VEGFR-2 staining was developed by tyramide amplification with Alexa Fluor-488 TSA kits (Molecular Probes). Cell nuclei were stained with VectorShield Mounting Medium containing DAPI (Vector Labs). VEGFR-2 and CD31 staining in tissues was quantitated by Photoshop (Adobe Photoshop version 5.0, Adobe Systems Incorporated, San Jose CA) analysis of images captured with an Axiovert 200 epifluourescent microscope and/or by high resolution scanning analysis with a Laser Scanning Cytometer (CompuCyte Corp. Cambridge, MA). In the first method, images were captured from at least five different 40× fields containing areas of high vascular density in each tumor section. The separate images of CD31 and VEGFR-2 staining were converted to tiff files and imported into Photoshop, where they were converted to gray scale and inverted so that positive staining appeared dark on a white background. The threshold function was adjusted to give binary black and white images that reflected the original staining pattern. The threshold setting was held constant for all images captured from all tumors and each image was analyzed with the histogram function to determine the percentage of the image area that contained black pixels as a readout for the amount of positive staining. In the second method, slides were scanned with a Laser Scanning Cytometer, whose red and green fluorescent photodetector channels were set at the same level for all tissue sections. Three areas with relatively homogeneous staining were chosen from low resolution scans of tumors to analyze at high resolution. Gates were set to exclude background staining in the red and green channels and the percentage of red only pixels, green only pixels, and red plus green pixels within each high resolution field were quantitated. These parameters provided readouts of total CD31 and VEGR-2 staining within a selected area and precise quantitation of the percentage of co-localized staining.

### 2.8. Statistics

All statistical analyses were performed with Number Cruncher Statistical Software (NCSS) version 2007 which includes routines to test datasets for the validity of assumptions of normal distribution and equal variance. Data that met these assumptions were analyzed by ANOVA and post-hoc comparisons were performed with Dunnett’s one-sided multiple comparison test. The *p*-values of individual pairwise comparisons were calculated from the *F*-distribution. Statistical comparisons of data that were not normally distributed were made by the distribution-free Kruskal-Wallis multiple comparison *Z*-value test. The slopes of linear regression curves calculated from the square roots of tumor volumes plotted against time served as estimates of tumor growth rates and were compared for differences by two sample *t*-tests. Kaplan-Meier survival curves were constructed and the logrank test was applied to test for differences in survival between groups. Comparisons between two proportions were made with Fisher’s or with Barnard’s one-sided exact test for which *p* < 0.05 were considered significant. Quantitative data are presented graphically as box plots, in which rectangles represent the middle 50% of the data points, the median is shown as a line in the rectangle, and whiskers represent the high and low observations within the dataset.

## 3. Results

### 3.1. SLT-VEGF Activates Caspases 3/7 and 8 in Endothelial Cells Overexpressing VEGFR-2 but Not in Line IV Cl 1 Melanoma Cells

**Figure 1:**
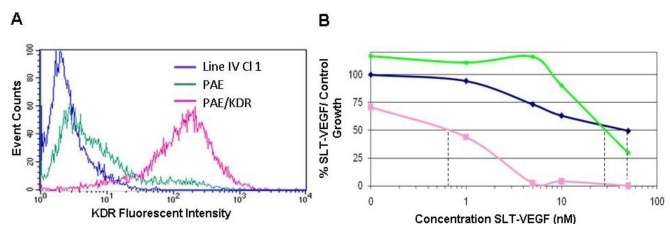
SLT-VEGF selectively targets VEGFR-2 expressing cells. High levels of VEGR-2 were detected on PAE/KDR cells (pink lines, **A**) by FACS analysis with a PE‑labeled monoclonal antibody specifically recognizing human VEGFR-2 (R & D Systems, Minneapolis, MN). Untransfected PAE cells (green lines) and human Line IV Cl 1 melanoma cells (blue lines) had negligible VEGFR-2 expression. When tested in a four day growth inhibition assay for sensitivity to SLT-VEGF, PAE/KDR cells had an IC_50_ that was roughly 70–100 times lower than those of PAE cells and Line IV Cl 1 cells (**B**).

We reported previously that SLT-VEGF is highly cytotoxic (IC_50_—0.2 nM) to PAE/KDR endothelial cells expressing ~2 × 10^5^ VEGFR-2/cells, and that its cytotoxicity was ~20-fold lower for PAE cells lacking VEGFR-2 [18]. Considering that tumor cells may express VEGF receptors [19,20,21], and that even non-receptor mediated SLT-VEGF toxicity might be cell-specific, we explored the sensitivity of Line IV Cl 1 melanoma cells to SLT-VEGF *in vitro*. We found that these cells are essentially negative for VEGFR-2 expression (Figure 1A) and their growth is inhibited by SLT-VEGF at a 100-fold higher IC_50_ of 70 nM (Figure 1B).

**Figure 2:**
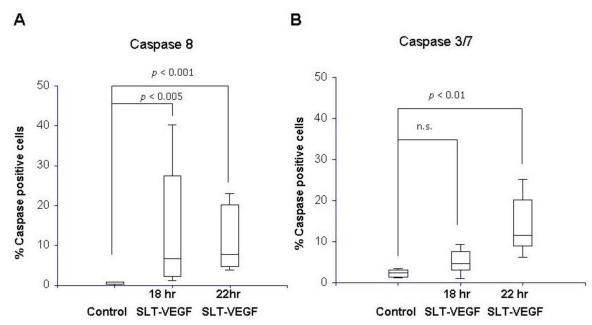
Caspase 8 and caspases 3/7 are activated by SLT-VEGF treatment of PAE/KDR cells. PAE/KDR cells treated with 26 nM SLT-VEGF had significant activation of caspase 8 (**A**) as measured by the percentage of caspase positive cells in the FLICA assay at 18 and 22 hours of incubation compared to untreated control cells. Caspase 3/7 (**B**) shows significant activation at 22 hours compared to control cells.

In agreement with growth inhibition data, SLT-VEGF activated caspases 8 and 3/7 in PAE/KDR cells but not in Line IV Cl 1 melanoma cells, as measured by fluorescence microscopy with FLICA 8 and FLICA 3/7 activated caspase detection reagents (Figure 2). As expected, activation of the apical caspase 8 was detected earlier in SLT-VEGF treated PAE/KDR cells than activation of effector caspases 3/7. Caspase 8 was activated at the 18 and 22 hour time points while caspase 3/7 activation was delayed until the 22 hour timepoint (all comparisons made by the Kruskal-Wallis multiple comparison test, α = 0.05).

### 3.2. SLT-VEGF Depletes Tumor Vasculature of VEGFR-2 Expressing Cells, but Does Not Inhibit Primary Tumor Growth

Line IV Cl 1 melanoma cells developed palpable tumors in NCR athymic mice one week after intradermal inoculation of 1 × 10^6^ cells into the flank. At that time mice were divided into treatment and control groups, which received twice weekly subcutaneous injections in 100 μL of injection saline of a total of five, 0.2 mg/kg doses of SLT-VEGF or saline vehicle alone prior to tumor excision.

**Figure 3:**
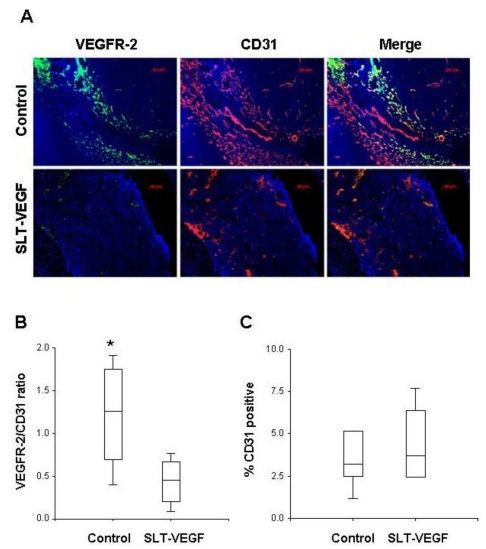
SLT-VEGF treatment results in selective depletion of VEGFR-2 expressing CD31^+^ tumor endothelial cells. (**A**) Immunofluorescent staining of frozen melanoma tumor sections with Alexa-488 labeled anti-VEGFR-2 antibodies (green) and Alexa-594 labeled anti-CD31 antibodies (red) revealed that most VEGFR-2 is coexpressed with CD31 positive endothelial cells. (**B**) VEGFR-2 and CD31 expression in melanoma tumors was quantitated by image analysis in Photoshop of six different 40× fields with high vascular density selected from each tumor. Dividing the percentage of image area positively stained with VEGFR-2 by that stained with CD31 yielded the VEGFR-2/CD31 ratio which was significantly (*: *p =* 0.013) reduced in SLT-VEGF treated tumors. (**C**) However, the total amount of CD31 staining in melanoma tumors was not significantly (*p* = 0.792) altered by SLT-VEGF treatment.

Immunofluorescent analysis of primary tumors revealed that most VEGFR-2 immunostaining colocalized with that of the pan-endothelial CD31 marker, but only a fraction of CD31^+^ endothelial cells expressed detectable amounts of VEGFR-2 (Figure 3A). SLT-VEGF treatment resulted in selective depletion of VEGFR-2 expressingCD31^+^ tumor endothelial cells (Figure 3A). Notably, those cells were particularly sensitive to SLT-VEGF treatment, leading to a 64% decrease in the ratio of VEGFR-2^+^ to CD31^+^ staining (Figure 3B) while there was not a significant decrease in total CD31 staining (Figure 3C).

**Figure 4:**
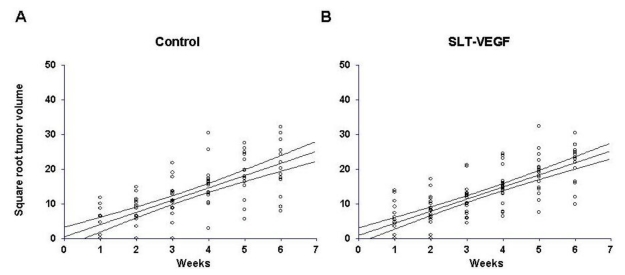
SLT-VEGF treatment does not significantly change the growth rate of orthotopic human melanoma tumors in NCR athymic mice. The growth rate of Line IV Cl 1 human melanoma tumors in control (*n* = 15, **A**) and SLT-VEGF (*n* = 20, **B**) treatment groups was estimated by linear regression on the square roots of tumor volumes against time. The average slopes ± 95% confidence intervals for tumor growth in control (3.52 ± 0.73) and SLT-VEGF treated (3.48 ± 0.58) mice were not significantly different. Both regression curves intersect the X-axis at or near the origin indicating that tumor cells inoculated in the skin of NCR athymic mice grow continuously from the time they are injected without a discernable lag period.

Despite significant depletion of VEGFR-2^+^/CD31^+^ tumor endothelial cells, SLT-VEGF treatment did not affect the growth of primary tumors (Figure 4), suggesting that in our model such cells do not play a critical role in maintaining angiogenesis once tumors have achieved a certain growth threshold. In part, it might be due to their low prevalence in tumor vasculature, as judged by the low uptake of VEGFR-2 mediated scVEGF/Cy fluorescent tracer in this tumor relative to others, such as those elicited with human U87 MG glioma and MDA-MB-231 breast carcinoma cell lines (data not shown).

### 3.3. SLT-VEGF Treatment Decreases the Incidence of Lung Metastases and Tumor Recurrence, and Improves the Overall Survival after Removal of Primary Tumor

**Figure 5:**
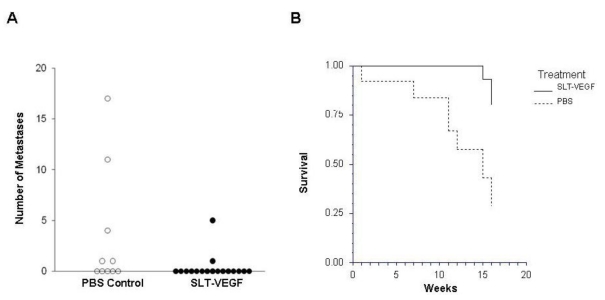
SLT-VEGF inhibits development of lung metastases and improves survival in NCR athymic mice following excision of Line IV Cl 1 human melanoma tumors. SLT-VEGF treated mice had a lower incidence of lung metastasis (*p =* 0.038, **A**) and survived significantly longer (*p* = 0.007, **B**) than control mice.

We reasoned that VEGFR-2^+^/CD31^+^ endothelial cells might play a more important role at the very early stages of tumor growth, when such cells appear in response to activation of a pro-angiogenic program in tumor cells, the so-called angiogenic switch [2]. We therefore explored effects of SLT‑VEGF treatment on metastatic dissemination and tumor recurrence, according to the following experimental protocol. Primary tumors were excised between six and seven weeks after tumor inoculation and then, after two weeks of recovery, mice received five additional injections of 0.2 mg/kg doses of SLT-VEGF or saline on a twice weekly schedule. We found that both the incidence (2/17 *versus* 5/11, *p* = 0.038 by Barnard’s one-sided exact test) and the average number of histologically confirmed lung metastases per mouse were significantly lower (*p* < 0.05 by the Kruskal‑Wallis *Z*‑value test, Figure 5A) in the SLT-VEGF group than in the control group. Interestingly, in the two SLT-VEGF treated mice that developed lung metastases the average diameter of tumor foci was larger (0.47 mm) than that of control mice (0.21 mm, *p* < 0.05). However the metastasis burden, here defined as the sum of the diameters of all foci in each set of lungs from mice with microscopic metastases, was not significantly different between SLT-VEGF treated and control animals (1.32 mm *vs.* 1.46 mm, *p* > 0.05). These results indicate that SLT-VEGF is effective in limiting the outgrowth of micrometastases and suggest that in instances where metastases do start growing they become insensitive to SLT-VEGF. In addition to its ability to reduce lung metastases, SLT-VEGF treatment also inhibited post-surgical tumor recurrence (0/15 in SLT-VEGF treated *vs.* 4/10 in control mice, *p* = 0.007) and improved survival (*p* = 0.002, Figure 5B).

### 3.4. SLT-VEGF Depletes Tumor Vasculature of VEGFR-2 Positive Cells, but Does Not Affect Tumor Growth, Metastatic Dissemination, Tumor Recurrence, and Overall Survival in a Mouse Host Whose Line IV Cl 1 Melanoma Tumors Have a Longer Dormancy Period

**Figure 6:**
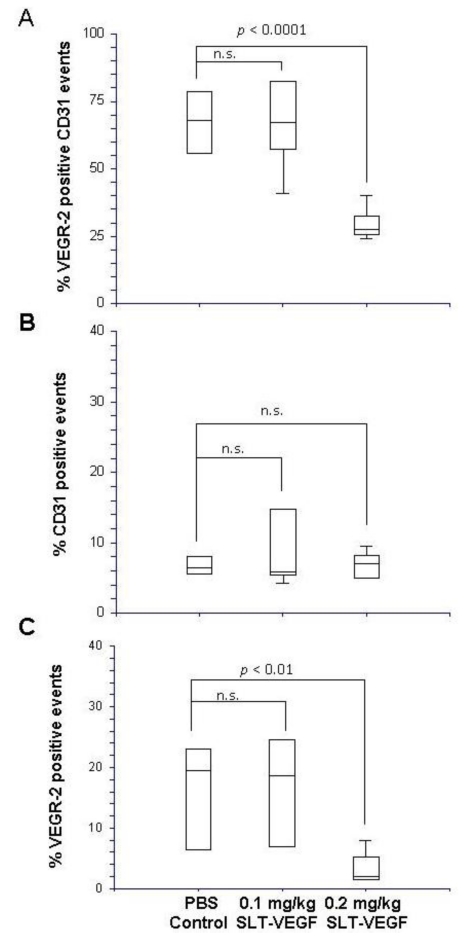
Reduction of VEGFR-2 expression on melanoma tumor vasculature by SLT‑VEGF is dose dependent. Line IV Cl 1 tumors grown in Balb/c nu/nu mice were excised after treatment with 0.1 mg/kg or 0.2 mg/kg doses of SLT-VEGF then frozen, sectioned and immunostained for their content of VEGFR-2 and CD31 expressing cells. In these experiments high resolution laser scanning cytometry was applied to precisely quantitate the percentages of cells co-expressing these markers. The percentage of VEGFR-2^+^/CD31^+^ events (**A**) and total VEGFR-2^+^ events (**C**) were significantly lower in the 0.2 mg/kg SLT-VEGF treatment group than in the 0.1 mg/kg treatment group and PBS control group, while total CD31^+^ events (**B**) were unchanged.

**Figure 7:**
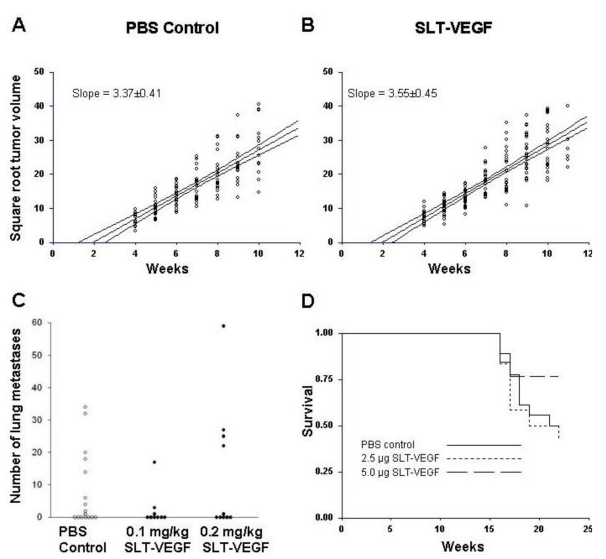
Tumor growth rate, incidence of lung metastases, and survival are not affected by SLT-VEGF treatment in a long tumor dormancy model of melanoma. Line IV Cl 1 cells did not develop measurable tumors until four weeks after inoculation into Balb/c nu/nu mice and displayed a two week dormancy period as indicated by the intersection with the X-axis of the regression curves calculated from the square roots of tumor volumes plotted against time (**A** and **B**). Growth rates of tumors as measured by slopes of the regression curves were not significantly different for SLT-VEGF treated (3.55 ±0.34, **B**) and PBS control (3.37 ±0.41, **A**) groups. The incidence of lung metastases in the 2.5 and 5 μg/injection dose groups of SLT-VEGF treated mice were not significantly different from the PBS control group (**C**). Although survival appears to be improved in mice in the 5 μg SLT‑VEGF injection dose group the difference between it and the PBS control group did not reach statistical significance by the logrank test (*p* = 0.198, **D**).

To assess additional factors affecting responsiveness to SLT-VEGF treatment we used orthotopic Line IV Cl 1 tumors grown in Balb/c nu/nu mice. In this mouse strain, tumors developed over a longer dormancy period, becoming palpable at four weeks after intradermal injection of 1 × 10^6^ cells/mouse, as opposed to one week for NCR athymic mice. Tumor-bearing Balb/c nu/nu mice were divided into saline control groups and treatment groups receiving SLT-VEGF at two different dose levels. Beginning at four weeks after tumor cell inoculation, mice in the latter two groups received a total of seven weekly injections of 0.1 mg/kg or 0.2 mg/kg doses of SLT-VEGF before surgical removal of primary tumors. After tumor excision, mice received an additional five 0.1 mg/kg or 0.2 mg/kg dose injections on a weekly schedule. At 0.2 mg/kg doses, VEGFR-2^+^/CD31^+^ endothelial cells were depleted from tumor vasculature (Figure 6A), total numbers of VEGFR-2^+ ^cells were reduced (Figure 6B) and total numbers of CD31^+^ cells were not changed (Figure 6C); similar to our experience with NCR athymic mice. However, in this model, SLT-VEGF did not affect the growth of primary tumors, post-surgical local recurrence of tumors, incidence of metastatic dissemination, or overall survival (Figure 7A–C). Thus, in a model with a longer dormancy period, SLT-VEGF did not produce any measurable improvements, despite selective cytotoxicity to VEGFR-2^+^/CD31^+^ endothelial cells at the 0.2 mg/kg dose level.

## 4. Discussion

We report here that SLT-VEGF, a fusion toxin that enters the cell via VEGF receptor mediated endocytosis, depletes VEGFR-2^+^/CD31^+^ endothelial cells from the vasculature of orthotopic Line IV Cl 1 melanoma tumors established in nude mice. This finding extends previously reported similar effects of SLT-VEGF on the tumor vasculature of subcutaneous PC3 and 4T1 tumors [11]. However, unlike those models, the growth of orthotopically inoculated Line IV Cl 1 melanoma cells is not affected by SLT-VEGF, indicating that either the level of depletion of VEGFR-2^+^/CD31^+^ cells was not sufficient to inhibit tumor progression or that the contribution of VEGFR-2^+^/CD31^+^ endothelial cells to tumor progression is not critical in this model.

We found that one million Line IV Cl 1 human melanoma cells inoculated intradermally into the flanks of NCR athymic mice formed palpable tumors 1 week after cell inoculation, whereas the same method and the same amount of cells inoculated in Balb/c nu/nu mice reproducibly did not produce similarly sized tumors until four weeks after inoculation. Even though the mechanism for such a difference remains unclear, we reasoned that we could use these two models, referred to here as short and long dormancy models, respectively, to study the effects of SLT-VEGF.

In clinical practice melanoma skin lesions are surgically removed, but patients are at risk of disease progression due to metastatic dissemination of tumor cells that are undetectable at the time of surgery. We therefore explored the effects of SLT-VEGF on development of metastasis in a model system where orthotopic melanoma tumors established in the skin have a high propensity to metastasize to the lungs [15]. We found that SLT-VEGF inhibits the incidences of lung metastases and tumor recurrence after removal of primary Line IV Cl 1 melanoma in NCR athymic mice (short dormancy model). These data are in agreement with the results obtained in an orthotopic model of pancreatic cancer, in which SLT-VEGF inhibited metastatic dissemination [13]. In contrast, we found no statistically significant difference in the incidence of lung metastases among the treatment groups in a long dormancy melanoma model.

These data indicate that even at this very early stage of tumor angiogenesis the contribution of VEGFR-2^+^/CD31^+^ endothelial cells might depend on the complex dynamics of tumor-host interactions. Indeed, as we have recently reported, SLT-VEGF treatment of a syngeneic murine 4T1 tumor started as early as four days after implantation of 2,000 cells/mouse resulted in a distinct separation of treated mice into “responders” and “non-responders”. Furthermore, an increase in SLT-VEGF dose changed the proportion of responders but not the degree of growth tumor inhibition [11].

We report here that SLT-VEGF works through initiation of caspase-dependent apoptosis in VEGFR-2 expressing cells. SLT holotoxin activates specifically caspases 8, 9, 3 and 6 [22,23]. We observed a significant level of caspase 3/7 and 8 activation in PAE/KDR cells, while no caspase activation was detected in the melanoma cells which express little or no VEGFR-2. These data, together with our previous work on activation of casapse 6 in PAE/KDR exposed to SLT-VEGF [18] indicate that, at least *in vitro*, SLT-VEGF activates caspases in the same manner as the holotoxin.

It should be noted that two other targeted toxins containing VEGF have been reported to date, diphtheria toxin (DT) and gelonin [24,25,26,27]. Although they provided a “proof-of-principle” that VEGF‑toxin fusion proteins work *in vivo*, their further development is doubtful for several reasons. First, the reported cytotoxicity of VEGF-DT to endothelial cells of normal vasculature (HUVEC) indicates that it would display high non-specific toxicity. In contrast, as we reported earlier and confirmed here, SLT-VEGF requires high levels of VEGFR-2 to exert cytotoxic effects [11,16,18]. Second, high immunogenicity and pre-existing immunity to DT is a serious obstacle for clinical development of VEGF-DT [28,29]. In contrast, SLT-VEGF induces only a low level anti-SLT serum response even after 3–5 consecutive injections [11]. Third, the low yield of VEGF-gelonin produced in *E. coli* (0.23 mg/L) is problematic for its pre-clinical development [27]. As recently reported, we have developed a scalable GMP-compatible procedure for production of SLT-VEGF at a level of 4–5 mg/L [11], which is a reasonable start for clinical development of SLT-VEGF.

In conclusion, our data show that SLT-VEGF is a selectively cytotoxic anti-angiogenic protein that improves survival and inhibits metastasis in a short dormancy model of orthotopic melanoma. The narrow “window of vulnerability” for treatment with SLT-VEGF raises the questions about the translational potential of this targeted toxin. However, recent research on mechanisms of action of anti‑angiogenic drugs that target VEGF/VEGFR signaling suggests a new opportunity for SLT–VEGF. Studies with several approved angiogenesis inhibitors indicate that they induce rapid vascular regression followed by revascularization, particularly at the edges of the tumor [30,31]. Since such revascularization supports continued tumor growth and metastatic dissemination, it is most likely responsible for the relatively low success rate of anti-angiogenic therapy. Our recent imaging studies in several tumor models indicate that revascularization is associated with a resurgence in the prevalence of VEGFR2^+^/CD31^+^ positive cells [30,31]. Preliminary results indicate that such revascularization could be inhibited by SLT-VEGF [11]. Experiments are now in progress to establish if combining angiogenesis inhibitors with SLT-VEGF might provide therapeutic benefits.
